# Imaging activity possibly signalling missed diagnostic opportunities in bladder and kidney cancer: A longitudinal data-linkage study using primary care electronic health records

**DOI:** 10.1016/j.canep.2020.101703

**Published:** 2020-06

**Authors:** Yin Zhou, Gary A. Abel, William Hamilton, Hardeep Singh, Fiona M. Walter, Georgios Lyratzopoulos

**Affiliations:** a Primary Care Unit, Department of Public Health and Primary Care, University of Cambridge, Cambridge, UK; b College of Medicine and Health, University of Exeter Medical School (Primary Care), Exeter, UK; c Center for Innovations in Quality, Effectiveness and Safety, Michael E. DeBakey Veterans Affairs Medical Center and Baylor College of Medicine, Houston, TX, USA; d Epidemiology of Cancer Healthcare and Outcomes (ECHO) Research Group, Department of Behavioural Science and Health, University College London, London, UK

**Keywords:** Bladder cancer, Kidney cancer, Early diagnosis, Imaging test, Diagnostic delay

## Abstract

**Introduction:**

Sub-optimal use or interpretation of imaging investigations prior to diagnosis of certain cancers may be associated with less timely diagnosis, but pre-diagnostic imaging activity for urological cancer is unknown.

**Method:**

We analysed linked data derived from primary and secondary care records and cancer registration to evaluate the use of clinically relevant imaging tests pre-diagnosis, in patients with bladder and kidney cancer diagnosed in 2012-15 in England. As pre-diagnostic imaging activity increased from background rate 8 months pre-diagnosis, we used logistic regression to determine factors associated with first imaging test occurring 4–8 months pre-diagnosis, considering that such instances may reflect possible missed opportunities for expediting the diagnosis.

**Results:**

1963 patients with bladder or kidney cancer had at least one imaging test in the 8 months pre-diagnosis. 420 (21%) of patients had their first imaging test 4–8 months pre-diagnosis, that being ultrasound, CT and X-ray in 48%, 43% and 9% of those cases, respectively. Factors associated with greater risk of a first imaging test 4–8 months pre-diagnosis were kidney cancer, diagnosis at stages other than stage IV, first imaging having been an X-ray, test requested by GP and absence of haematuria before the imaging request.

**Conclusion:**

About 1 in 5 patients with urological cancers receive relevant first imaging investigations 4–8 months prior to diagnosis, which may represent potential missed diagnostic opportunities for earlier diagnosis.

## Highlights

• Suboptimal imaging test use may represent missed opportunities for more timely diagnosis of bladder and kidney cancer.
• Our novel linked dataset described patterns of imaging test use and predictors of a first imaging test use in patients with these cancers.
• 1 in 5 patients received a longer than average time to diagnosis of 4–8 months after a first imaging test.
• Patients with less specific symptoms, and with kidney cancer were more likely to receive a first imaging test 4–8 months before diagnosis.

## 1 Introduction

Timely diagnosis of cancer is associated with better clinical and patient reported outcomes [1,2]. In the United Kingdom (UK), a number of early diagnosis initiatives have been implemented over the last 12 years [3].

Patterns of pre-diagnostic healthcare utilisation may indicate opportunities for expediting the diagnosis of cancer; these could include increase in the background rate of consultations, prescriptions and laboratory test use, long before the immediate pre-diagnosis period [[4], [5], [6], [7], [8], [9], [10], [11]]. While it is plausible that the rate of imaging activity could also increase long before the diagnosis of cancer [12,13], we are unaware of such evidence in patients with bladder and kidney cancer. Such events may represent missed opportunities for more timely diagnosis of cancer. Possible scenarios include: normal findings leading to ‘false reassurance’ and diagnostic closure where investigations ought to have continued; and abnormal findings either not being appropriately enacted upon, scheduling delays or other system factors delaying planned subsequent assessment [14].

In the UK, about 10,000 and 12,500 patients are diagnosed with bladder and kidney cancer respectively every year (hereafter referred to as urological cancer, unless otherwise specified) [15]. While a small proportion of small kidney cancers diagnosed via imaging might represent incidental findings in asymptomatic individuals [16], imaging tests such as ultrasound or computed tomography (CT) have a role in investigating symptomatic patients with suspected urological cancer [[17], [18], [19], [20]]. Although general practitioners (GPs) may have direct access to some imaging tests (such as ultrasound), delays relating to the scheduling, performing and reporting of these tests may occur.

Given this background, we aimed to describe the patterns of pre-diagnostic imaging test use and predictors of a first imaging test in bladder and kidney cancer patients occurring several months pre-diagnosis. This type of analysis could help estimate the frequency of possible missed opportunities related to the use of imaging investigations for a more timely diagnosis of urological cancer, and factors that may be associated with them.

## 2 Methods

### 2.1 Data sources

We used primary care data from the Clinical Practice Research Datalink (CPRD) that provides patient-level linkage to data from the National Cancer Registration Analysis Services (NCRAS), Hospital Episode Statistics Diagnostic Imaging Dataset (HES DID) and Index of Multiple Deprivation quintiles (deprivation indices defined for small geographies) [21].

The CPRD contains primary care data from about 7% of GP practices in England, Wales and Scotland, with coverage that is approximately representative of the UK population [22]. About 75% of practices in England have consented to data linkage with other data sets and our study is restricted to those practices [22]. NCRAS data contains detailed tumour level information, including cancer site and date of diagnosis. HES DID contains imaging tests that are performed in English National Health Service (NHS) hospitals, including information on imaging modality, imaged body sites, referral source (e.g. primary/specialist care) and date, referral receipt date, and imaging and reporting dates.

### 2.2 Study population

A comprehensive list of Read diagnosis codes for bladder and kidney cancer were provided to CPRD to extract the cohort, concordant with prior literature [23,24]. Included patients were aged 25 years and over at diagnosis of cancer, with a first-ever recorded bladder or kidney cancer between 1st April 2012 and 31st December 2015. We supplemented CPRD cases with additional cases identified using ICD-10 cancer codes from NCRAS only, and used the NCRAS diagnosis and date where discrepancies existed. Cancers were sub-divided into bladder, kidney or upper urinary tract urothelial cell cancer.

### 2.3 Imaging types

We used the National Interim Clinical Imaging Procedure codes to determine all imaging tests performed in our patient cohort in the 12 months before their cancer diagnosis. Although information was available on seven modalities (x-ray, ultrasound, computed tomography (CTs), magnetic resonance imaging (MRI), fluoroscopy, image-guided endoscopy and nuclear medicine) hereafter we focus on X-ray, ultrasound and CT imaging events, as the most relevant modalities for investigation of possible urological cancer and as use of other modalities in our cases was very infrequent.

A clinician (YZ) grouped each imaging modality by body site into a) urinary tract-related, b) abdomen (without specific mention of urinary tract organs), and c) other body sites. Imaging tests for other body sites were a priori excluded to minimise potential bias for requests for unrelated reasons, particularly regarding X-ray activity (Appendix A).

The full list of diagnosis, imaging codes, corresponding modalities and body sites is available from the authors on request.

### 2.4 Descriptive statistics

We initially estimated the imaging rate by month (number of imaging tests / number of patients in the cohort) performed in the 12 months before diagnosis, and using Poisson regression, we identified the likely inflection point at which there was evidence that activity changed from a background rate (Appendix C). This was around 6 months pre-diagnosis for CT, 7 months for ultrasound and 8 months for X-ray. For consistency, and so as to not ignore any relevant imaging, an 8-month cut-off was used for all modalities. We then found the first test in the year before diagnosis, and restricted all subsequent descriptive analyses to patients with their first test performed up to 8 months pre-diagnosis.

### 2.5 Sub-analysis

We performed crude, then adjusted, logistic regression analyses to examine the association between patient, imaging and tumour variables, and an index test having occurred between 4–8 months compared with one occurring 0–3 months pre-diagnosis. We regarded 3 months to be a conservative cut-off for the duration which one could expect a patient who had an initial imaging test to be diagnosed with cancer.

Patient variables included gender, age group, and presence/absence of haematuria before the first imaging request and up to 2 years pre-diagnosis (based on CPRD records); Index of Multiple Deprivation quintile; and ethnicity (based on HES records). Haematuria was defined using clinical Read codes used in previous studies [23,24]. The imaging characteristics included imaging modality and source of imaging referral (derived from the DID dataset), and cancer variables (cancer site, stage at diagnosis, year of diagnosis) were from NCRAS data.

All analyses were performed using STATA v15.

## 3 Results

2,971 urological cancer patients diagnosed between 1st April 2012 and 31st December 2015 had linked CPRD, NCRAS and HES DID data, of whom 2,261 (76%) had at least one imaging test in the 12 months pre-diagnosis. After exclusions (Appendices A and B), a final sample of 1988 patients was included in subsequent analyses. Most patients had one (39%) or two (35%) scans in the year pre-diagnosis; 3.5% had 5 or more scans.

### 3.1 Imaging rate

**Fig. 1:**
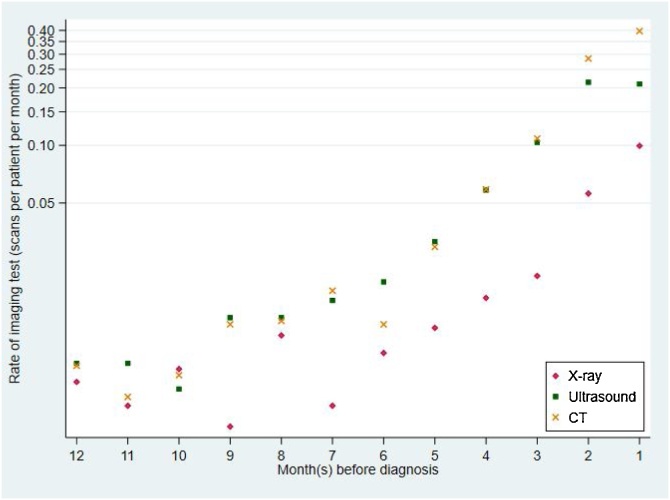
Incidence rate of imaging test for each of the three imaging modalities (logarithmic scale).

Imaging rates for all three modalities increased towards diagnosis, particularly so for ultrasound and CT tests compared with X-rays (Fig. 1). Poisson regression provided evidence for imaging activity increasing from background rates at about 6 months pre-diagnosis for CT, 7 months pre-diagnosis for ultrasound and 8 months for X-ray (Appendix C). We therefore used 8 months pre-diagnosis as the earliest pre-diagnosis time point during which relevant imaging tests could possibly indicate that a potential missed diagnostic opportunity could have occurred.

A Descriptive statistics

### 3.2 First imaging test

1971 patients had their first imaging tests in the 8 months prior to diagnosis; among those 11% had an X-ray, 48% an ultrasound, and 41% a CT as their first requested test (Table 1).

**Table 1:** Frequency of urological cancer patients’ first imaging tests for each month before diagnosis for all imaging modalities between 0 and 8 months pre-diagnosis.

Month pre-diagnosis	X-ray			Ultrasound			CT			any of x-ray/ ultrasound/ct	
	N	%	Cum. %^a^	N	%	Cum. %^a^	N	%	Cum. %	N	%
**1**	66	32	3	249	26	13	334	41	17	649	33
**2**	50	24	6	311	33	29	253	31	30	614	31
**3**	23	11	7	173	18	37	84	10	34	280	14
**4**	13	6	8	94	10	42	49	6	37	156	8
**5**	14	7	8	48	5	45	31	4	38	93	5
**6**	12	6	9	31	3	46	11	1	39	54	3
**7**	9	4	10	25	3	47	24	3	40	58	3
**8**	20	10	11	20	2	48	19	2	41	59	3
**Total**	207			951			805			1,963	

a Cumulative percentage against whole cohort n = 1,963.

1,543 (79 %) patients had their first imaging test 0–3 months pre-diagnosis, and 428 (21 %) patients 4–8 months pre-diagnosis; 48%, 43% and 9% of the 4–8 month group had ultrasound, CT scans and X-rays respectively.

### 3.3 Imaging request source

**Fig. 2:**
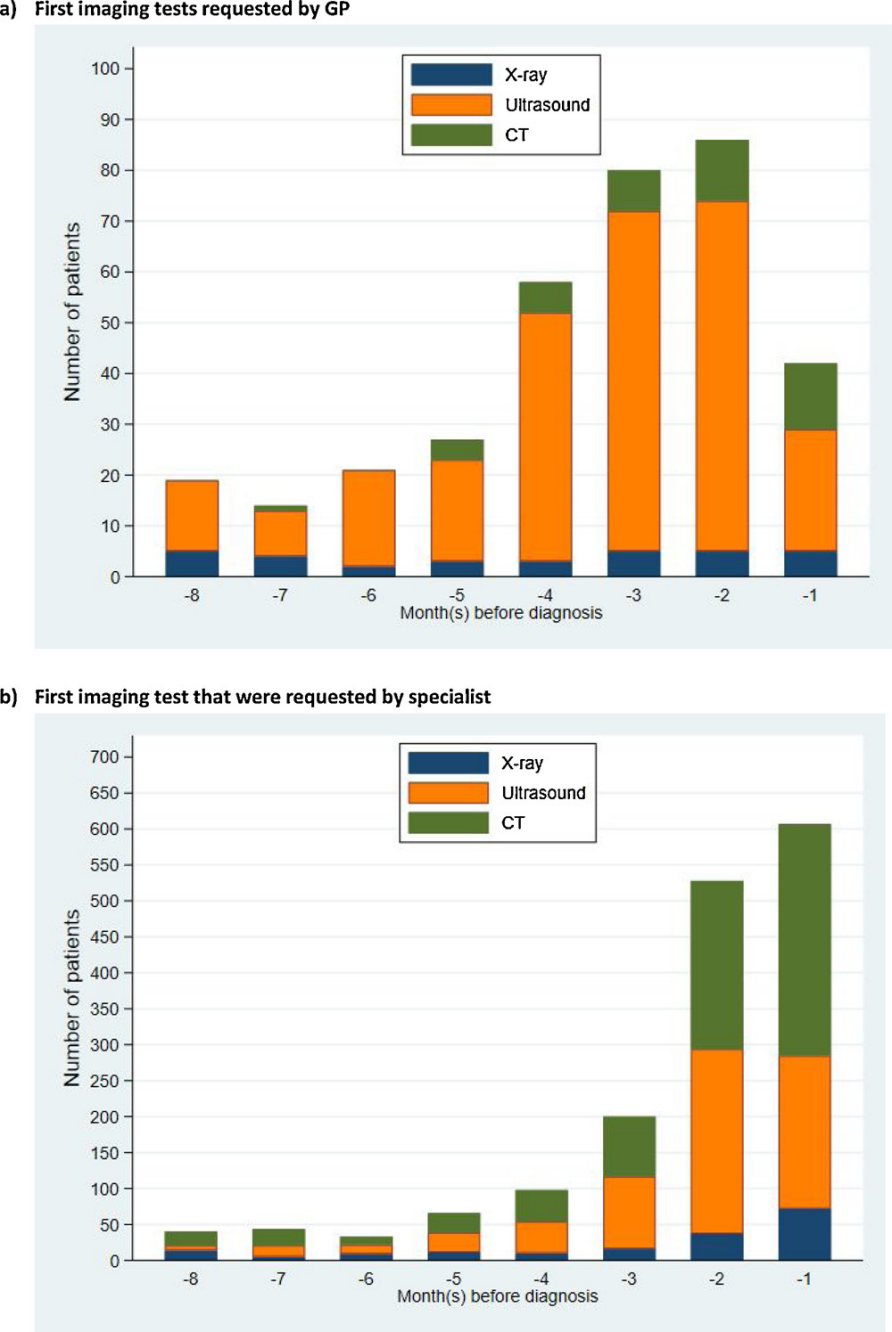
Number of patients who had each of the three modalities as their first imaging test in the 8 months before diagnosis by imaging request source (*NB. Please note difference in y-axis scale between figures*).

Excluding imaging tests that might relate directly to the cancer diagnosis itself (those <1month pre-diagnosis), 305/1,314 (23%) patients had a first imaging test requested by a GP, and 76% by specialists in the 18 months pre-diagnosis. The type of requested tests differed by source: 81 % of GP-requested imaging tests related to an ultrasound, while in contrast the corresponding figure for requests by specialists was 45%. The increase in the use of imaging test in the months leading up to cancer diagnosis was mostly for ultrasound in GP-referred cases, but the increase was similar for both ultrasound and CT scans in specialist-referred cases (Fig. 2).

B Additional analyses

Univariable and adjusted analyses provided concordant evidence in identifying factors associated with first imaging test occurring 4–8 months pre-diagnosis (Table 2).

**Table 2:** Results of logistic regression of association between patient, imaging and cancer variables and the odds of having a first imaging test between 4-8 months (compared to 0-3 months) pre-diagnosis.

Variable	TOtal	Patients with early test		Crude OR (95 % CI)	p-value	Adjusted OR^1^ (95 % CI)	p-value
	N	N	%				
Gender							
Male	1,345	278	20.7	Reference	0.247	Reference	0.613
Female	618	142	23.0	1.14 (0.91, 1.44)		0.94 (0.73, 1.21)	
Age Group							
<35	10	4	40.0	2.89 (0.76, 10.95)	0.315	2.74 (0.62, 12.20)	0.248
35-44	49	8	16.3	0.85 (0.36, 2.01)		0.87 (0.34, 2.23)	
45-54	144	27	18.8	Reference		Reference	
55-64	317	76	24.0	1.37 (0.84, 2.23)		1.72 (1.01, 2.94)	
65-74	621	140	22.5	1.26 (0.80, 2.00)		1.56 (0.95, 2.57)	
75-84	573	121	21.1	1.16 (0.73, 1.85)		1.63 (0.98, 2.71)	
85+	249	44	17.7	0.93 (0.55, 1.58)		1.27 (0.71, 2.26)	
Ethnicity							
White	1,878	406	21.6	Reference	0.172	Reference	0.318
Asian	21	5	23.8	1.13 (0.41, 3.11)		1.41 (0.48, 4.10)	
Black	14	5	35.7	2.01 (0.67, 6.04)		1.43 (0.43, 4.74)	
Mixed	4	0	0.0	Omitted		Omitted	
Other/ Unknown	42	4	9.5	0.38 (0.14, 1.08)		0.39 (0.13, 1.18)	
IMD							
1	461	96	20.8	1.12 (0.77, 1.63)	0.207	1.34 (0.89, 2.02)	0.432
2	487	103	21.1	1.14 (0.79, 1.65)		1.34 (0.90, 2.01)	
3	418	84	20.1	1.07 (0.73, 1.57)		1.19 (0.78, 1.80)	
4	324	85	26.2	1.51 (1.02, 2.23)		1.47 (0.96, 2.24)	
5	273	52	19.0	Reference		Reference	
Haematuria							
No	1,016	314	30.9	Reference	<0.001	Reference	<0.001
Yes	947	106	11.2	3.55 (2.79, 4.52)		3.02 (2.32, 3.95)	
Modality							
X-ray	207	68	33.3	2.45 (1.74, 3.46)	<0.001	2.89 (1.97, 4.22)	<0.001
USS	951	218	22.9	1.49 (1.17, 1.89)		1.34 (1.01, 1.76)	
CT	805	134	16.6	Reference		Reference	
Gp-referred							
No	1,616	280	17.3	Reference	<0.001	Reference	<0.001
Yes	347	140	40.3	3.23 (2.51, 4.14)		2.51 (1.88, 3.36)	
Cancer site							
Bladder	1,197	199	16.6	Reference	<0.001	Reference	<0.001
Kidney	680	192	28.2	1.97 (1.57, 2.47)		1.75 (1.29, 2.37)	
UUTUCC	86	29	33.7	2.55 (1.59, 4.09)		2.85 (1.67, 4.85)	
Stage							
0	415	74	17.8	Reference	<0.001	Reference	<0.001
1	378	102	27.0	1.70 (1.21, 2.39)		1.07 (0.71, 1.60)	
2	155	24	15.5	0.84 (0.51, 1.40)		0.65 (0.37, 1.11)	
3	160	40	25.0	1.54 (0.99, 2.38)		0.86 (0.51, 1.45)	
4	247	31	12.6	0.66 (0.42, 1.04)		0.29 (0.17, 0.50)	
Unknown	338	78	23.1	1.38 (0.97, 1.97)		1.08 (0.72, 1.62)	
Missing	270	71	26.3	1.64 (1.14, 2.38)		1.02 (0.66, 1.60)	

Abbreviations: CI= confidence interval; CT=computed tomography; IMD= index of multiple deprivation; N= number of patients; OR = odds ratio; USS=ultrasound; UUTUCC= upper urinary tract urothelial cell carcinoma.

All p-values based on joint Wald test of categorical variables.

1 Model also adjusted for year of diagnosis, p-value not significant (results not shown).

In the adjusted analyses, patients without haematuria recorded before the first imaging test had an increased odds of having a first imaging test 4–8 months pre-diagnosis compared to those with haematuria (adjusted OR 3.02 (CI 2.32–3.95), p < 0.001). Those diagnosed with kidney or urothelial cell cancer had 2- and 3-fold greater odds, respectively, of having a test 4–8 months pre-diagnosis compared to bladder cancer patients (adjusted OR compared with bladder cancer: 2.85 (CI 1.67–4.85) for UUTUCC; 1.75 (CI 1.29–2.37) for kidney cancer, p < 0.001). Having an X-ray compared to ultrasound and CT as the first imaging test (adjusted OR 2.89 (CI 1.97–4.22), p < 0.001 X-ray vs CT), and having a GP-requested (vs specialist-requested) first imaging test (adjusted OR 2.51 (CI 1.88–3.36), p < 0.001 GP vs non-GP) were also associated with greater likelihood of having a first imaging test 4–8 months pre-diagnosis. Patients with stage 4 cancer were least likely to have had a first imaging test 4–8 months pre-diagnosis (adjusted OR 0.29 (CI 0.17-0.50), p < 0.001 Stage 4 vs 0).

Given that imaging tests are more likely to be relevant in the context of kidney compared to bladder cancer (where cystoscopy also plays a major role in the diagnostic pathway), we examined the frequencies of imaging type by cancer site in patients with a first imaging test between 4–8 months pre-diagnosis, and the number of patients who had no ultrasound or CT scans performed at any point after an initial imaging test (Table 3).

**Table 3:** Number of patients who had a first imaging test 4-8 months pre-diagnosis by cancer site and imaging modality.

	Bladder		Kidney		UUTUCC		Total
	N	%	N	%	N	%	
**First imaging test 4–8 months pre-diagnosis**	199	47.4	192	45.7	29	6.9	420
***Patients with the following imaging test***							
**Ultrasound**	154	50.8	130	42.9	19	6.3	303
**CT**	121	37.1	179	54.9	26	8.0	326
***Patients with no imaging test***							
**No ultrasound**	45	38.5	62	53.0	10	8.5	117
**No CT**	78	83.0	13	13.8	3	3.2	94
**No ultrasound or CT**	4	50.0	3	37.5	1	12.5	8

Abbreviation: CT = computed tomography; N = number; UUTUCC = upper urinary tract urothelial cell cancer.

Among urological cancer patients with an initial imaging test 4–8 months pre-diagnosis, 47%, 46% and 7% were subsequently diagnosed with bladder, kidney and upper tract urothelial cancer patients respectively. While comparing imaging modality, more than half of patients whose first test was an ultrasound were subsequently diagnosed with bladder cancer. In contrast, the majority of patients with an initial CT scan in the 4–8 months pre-diagnosis were subsequently diagnosed with kidney cancer (Table 3).

## 4 Discussion

We found that increased imaging activity occurs in many patients with urological cancer as early as 8 months before diagnosis. About 1 in 5 of these patients had a first imaging test between 4–8 months pre-diagnosis, representing a ‘diagnostic window’ period which might have led to earlier diagnosis. Factors associated with lower specificity of presentation were associated with increased likelihood of imaging activity 4–8 months pre-diagnosis.

Our findings are consistent with existing literature reporting increasing healthcare utilisation (including of diagnostic tests) in the few months prior to cancer diagnosis [[4], [5], [6],^10^,^25^]. The increase in GP-requested ultrasounds but not GP-requested CTs during in the 8 months pre-diagnosis likely reflects the availability of direct-access tests for ultrasound, but not for CT, to GPs in the English NHS.

1 in 5 patients had an imaging test that did not lead to a diagnosis until 4–8 months later. Potential delays can occur during the testing phase (i.e. from test request to test performance and reporting) but we found that the overall test interval from a request to reporting was generally short (median of 10 days, Appendix D). Delays outside the testing phase (i.e. from test reporting to diagnosis) could reflect the ordering of a less appropriate first/subsequent test (pre-analytical test phase), or missed/ delayed follow-up of a positive test result (post-analytical test phase) (Box 1) [26]. During the post-analytical phase, inaccurate, missed, or delayed follow-up of test results are common [27,28].

Box 1 **Potential causes of delay in test-to-diagnosis interval relating to the use of test.**

• Pre-analytical delays:
  o Inappropriate (to the clinical picture) test ordered due to interpretation or clinical reasoning errors
  o Test phase delays:
    ▪ Test scheduling delay
    ▪ Patient factors: postponing test
• Analytical delays:
  o Incorrect reporting of result (false negative or false positive)
• Post-analytical delay:
  o Missed or delayed follow-up

Alt-text: Box 1

Patients without haematuria (an alarm symptom that forms part of the presenting picture in about 70% of cases with bladder cancer, but less than a quarter of patients with kidney cancer [23,24]); and those subsequently diagnosed with kidney cancer were more likely to be at risk of a potential delayed diagnosis compared to those with haematuria and subsequently diagnosed with bladder cancer. This supports previous evidence that patients with non-specific symptoms, and ‘harder-to-suspect’ cancers (i.e. those where only a small percentage of patients present with symptoms of relatively high predictive value, in this instance kidney compared to bladder cancer), are more likely to be associated with diagnostic delay [29]. Our findings suggest in particular that patients with kidney cancer are more likely to have an initially non-specific or insensitive imaging test, or the imaging result may be challenging to interpret, leading to possible diagnostic delay after an initial imaging test. Patients with Stage 4 cancer are likely to present in serious clinical condition, prompting fast investigative action leading to a shorter time to diagnosis. Having an X-ray as an initial imaging test is associated with a longer time to diagnosis, compared to ultrasound and CT, as it has limited diagnostic accuracy in urological cancer. The first imaging tests performed 4–8 months pre-diagnosis were more likely to be GP-requested, probably due to potential delays in the scheduling, follow-up and referral processes after an abnormal direct-access imaging test arranged from primary care.

In patients subsequently diagnosed with bladder cancer, about 1 in 2 and 1 in 3 had an initial ultrasound and CT respectively between 4–8 months pre-diagnosis. In these patients, delays in a cystoscopy referral, or in carrying out the cystoscopy, could be likely explanations for the prolonged interval to diagnosis, although false reassurance from a false negative imaging test could also be possible reasons. However, about 55% of cancer patients with a CT and 43% of those with an ultrasound carried out 4–8 months pre-diagnosis were subsequently diagnosed with kidney cancer. For this group of patients, cystoscopy referral/scheduling delays, while possible (e.g. if the wrong urological site is suspected), are nonetheless less likely as, in most of those cases, it can be assumed that the presenting symptoms would have not being pointing to bladder cancer. Diagnostic delays in such cases might arise from issues during the analytical (test performance, reporting), and/or the post-analytical test phase (subsequent interpretation, scheduling of referrals or additional investigations).

### 4.1 Strengths and limitations

To our knowledge, this is the first study to describe pre-diagnostic imaging activity in urological cancer patients. We use a novel linked population-based dataset in a representative population, paving the way for exploring potential missed diagnostic opportunities in these patients.

DID contained patient-level information on the exact imaging test performed, allowing us to consider a ‘relevant’ imaging test depending on body site, within a time period that we have detected the imaging activity to be different from background activity. We therefore minimised any bias introduced from irrelevant tests performed in the cohort. We regarded the increase in imaging activity during this 0–8 month pre-diagnostic period as a response to relevant (to the subsequently diagnosed cancer) clinical symptoms or signs, and assumed that any potential cancer significant enough to have caused these clinical symptoms/signs would also be detectable by imaging, or that in the context of a negative test, alternative effective diagnostic strategies could have been pursued. These assumptions which underpin the logic model for our analysis are reasonable, but not certainly applicable to all patients.

Given the lack of availability of imaging test results in the DID source, and inability to examine the full medical records of these patients, we are not able to confidently infer whether among cases with imaging test 4–8 months pre-diagnosis, there was a missed diagnostic opportunity in their pathway, only that this could have been possibly the case. In addition, our source data collected by NHS Digital (the Diagnostic Imaging Dataset) is a priori excluding non-NHS scans (e.g. those carried out in private hospitals). The lack of data on private imaging tests performed may lead to slight underestimation of the true burden of imaging tests performed 4–8 months pre-diagnosis that may represent missed opportunities.

### 4.2 Implications

There is increasing evidence that optimisation of the testing phase during the diagnostic process is crucial to improving diagnostic quality and safety, and this includes being able to maintain a vigilant outlook and avoiding premature diagnostic closure when no firm cause of symptoms can be found. Further, a test needs to be followed-up and acted upon after it has been ordered and performed to establish the findings [30]. Better communication on how to receive and follow-up the results of tests includes the engagement of patients, primary and secondary care clinicians [26,31]. For example, patient portals allowing access to test results are increasingly being advocated to encourage patient engagement in their own test management and results follow-up [32]. Research into electronic triggers integrated into computer systems to remind clinicians to follow-up abnormal results has shown promising results in the United States, such triggers being able to correctly identify potential missed or delayed follow-up of abnormal test results in up to 60 % of the cases [33].

### 4.3 Conclusions

We found that diagnostic imaging activity increased from as early as 8 months before a urological cancer diagnosis, indicating that ‘signals’ to expedite the diagnosis of cancer may be detectable in up to 1 in 5 patients. Patients with less specific clinical features were more likely to have an early imaging test 4–8 months pre-diagnosis. The findings provide proof of concept that missed diagnostic opportunities, including relating to the use of imaging tests, may occur in many patients with urological cancers, and should stimulate additional inquiry.

## Authorship contribution

YZ, GAA, FMW and GL initiated, planned and designed the study. YZ had full access to all the data in the study, and conducted the data acquisition, management and analysis. GAA provided the statistical input for the data analysis. YZ drafted the manuscript, all authors interpretated the study results and critically revised the manuscript.

## Funding

This work was supported by the NIHR School for Primary Care Research (FR13/Grant Ref 346). YZ is funded by a Wellcome Trust Primary Care Clinician PhD Fellowship (203921/Z/16/Z). This research is linked to the CanTest Collaborative, which is funded by 10.13039/501100000289Cancer Research UK [C8640/A23385], for which FMW and WH are Co-Directors, GL an Associate Director and GAA and HS Collaborators. GL is supported by a Cancer Research UK Advanced Clinician Scientist Fellowship Award (C18081/A18180). HS is supported by the VA Health Services Research and Development Service (Presidential Early Career Award for Scientists and Engineers USA 14-274), the VA National Center for Patient Safety, the Agency for Health Care Research and Quality (R01HS022087), the 10.13039/100000936Gordon and Betty Moore Foundation and the Houston VA HSR&D Center for Innovations in Quality, Effectiveness and Safety (CIN 13-413).

## CRediT authorship contribution statement

**Yin Zhou:** Conceptualization, Methodology, Validation, Formal analysis, Data curation, Writing - original draft, Funding acquisition. **Gary A. Abel:** Methodology, Validation, Formal analysis, Writing - review & editing. **William Hamilton:** Writing - review & editing, Supervision. **Hardeep Singh:** Writing - review & editing, Supervision. **Fiona M. Walter:** Conceptualization, Methodology, Writing - review & editing, Supervision, Funding acquisition. **Georgios Lyratzopoulos:** Conceptualization, Methodology, Writing - review & editing, Supervision, Funding acquisition.

## Declaration of Competing Interest

None.

## Figures and Tables

**Table:** 

Modality and body site	Frequency	Percentage	Cumulative percentage
Xray			
Urinary tract	60	2.41	2.41
Abdomen/ pelvis	466	18.72	21.13
Other sites	1,963	78.87	100
Total	2,489	100	
USS			
Urinary tract	1,364	76.46	76.46
Abdomen/ pelvis	126	7.06	83.52
Other sites	294	16.48	100
Total	1,784	100	
CT			
Urinary tract	1,074	38.3	38.3
Abdomen/ pelvis	1,103	39.34	77.64
Other sites	627	22.36	100
Total	2,804	100	
MRI			
Urinary tract	121	29.09	29.09
Abdomen/ pelvis	65	15.63	44.71
Other sites	230	55.29	100
Total	416	100	
Fluoroscopy			
Urinary tract	135	40.3	40.3
Abdomen/ pelvis	60	17.91	58.21
Other sites	140	41.79	100
Total	335	100	
endoscopy			
Urinary tract	17	65.38	65.38
Abdomen/ pelvis	7	26.92	92.31
Other sites	2	7.69	100
Total	26	100	
Nuclear medicine			
Urinary tract	66	29.07	29.07
Abdomen/ pelvis	109	48.02	77.09
Other sites	52	22.91	100
Total	227	100	

**Figure:**
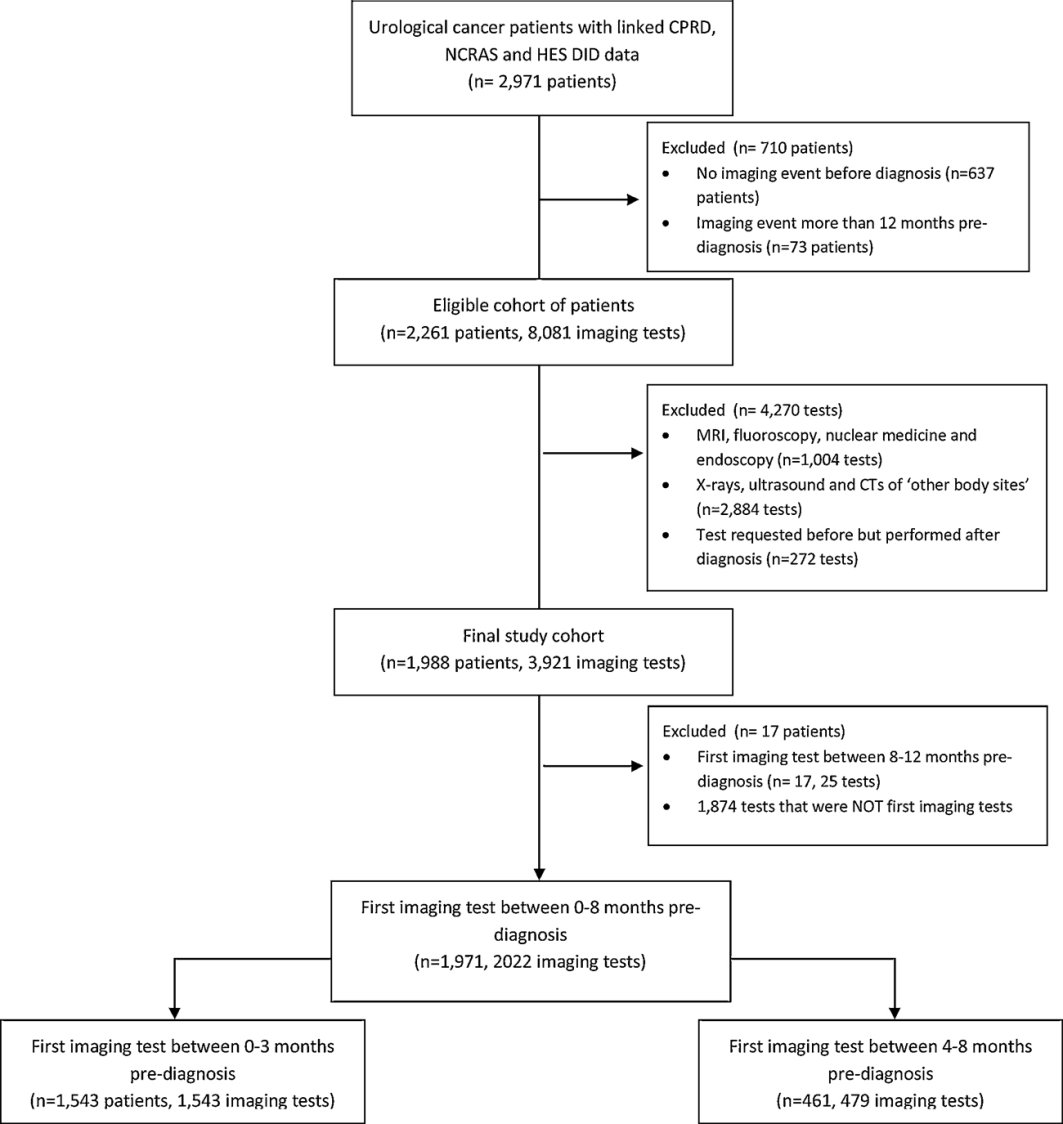


**Table:** 

Month before diagnosis	Log likelihood		
	X-ray	USS	CT
**4**	−32.777	−69.599	−67.229
**5**	−29.339	−43.949	−40.375
**6**	−28.113	−35.628	−40.189*
**7**	−29.426	−34.434*	−41.535
**8**	−29.090*	−36.428	−45.151
**9**	−30.153	−37.766	−47.888
**10**	−30.677	−41.805	−51.862
**11**	−31.358	−44.651	−56.032

**Fig. A1:**
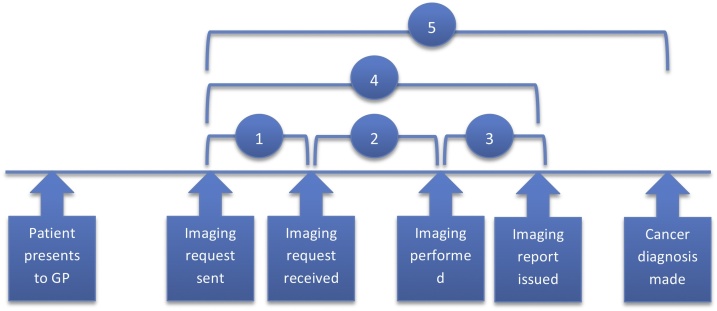
Schematic representation of the various test intervals. Interval 1: request interval; 2: scheduling interval; 3: reporting interval; 4: test interval; 5: test-to-diagnosis interval.

**Table:** 

Time interval	Test phase	No. of Patients	Mean (days)	InterQuartile Range (days)				
				10%	25%	50%	75%	90%
Request sent to diagnosis	Test to diagnosis	1,314	84	36	44	64	105	170
Request sent to report	Test interval	1,314	17	0	2.5	10	21	36
Request sent to test	Total pre-analytical	1,314	13	0	1	8	18	34
Request sent to received	Pre-analytical (admin)	1,314	1	0	0	0	0	2
Request received to test	Pre-analytical (scheduling)	1,314	15	0	1	7	17	33
Test to report	Post-analytical (reporting)	1,314	2	0	0	0	2	6

**Figure:**
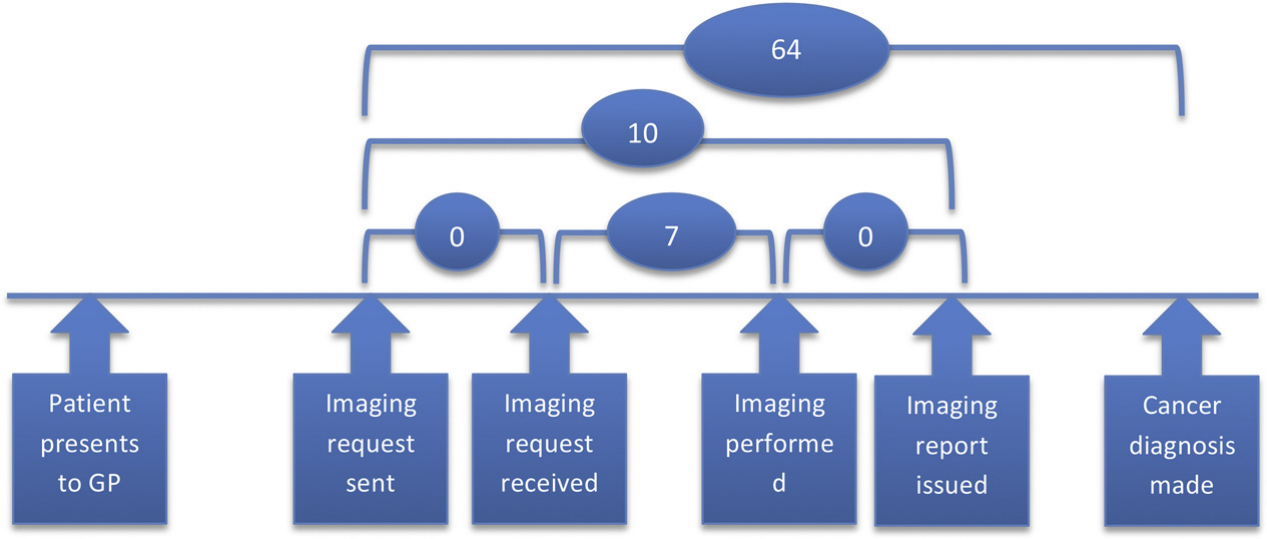


## Appendix C Poisson Regression estimates for inflection point for each of the three imaging modalities

In order to estimate the time relative to diagnosis that imaging frequency changed from a background rate, we utilised a series of Poisson regression models exploring the different possible inflection points. For each imaging mode separately, a model was run for each inflection point from 4 months to 11 months. In each case the monthly count of imaging was modelled by including a constant term, to account for the background rate, and a variable equal to the number of months between the inflection point and the month of interest for months closer to diagnosis than the inflection point and equal to zero otherwise. Data from 1 and 2 months prior to diagnosis were ignored for this analysis due to a levelling off in the rate of some imaging close to diagnosis. The log-likelihood for each model was recorded and the lowest value was taken to indicate the best fit to the data. For consistency, and so as to not ignore any relevant imaging, an 8 month cut-off was used for all modalities, being the longest time period across the three imaging modalities.

* Indicates likely inflection point

## Appendix D Test request to reporting interval (total test interval)

We defined the various testing intervals using dates of the testing activities reported in HES DID, as follow:

a) Request interval: time taken from the imaging request being sent to being received (interval 1)
b) Scheduling interval: time from imaging request being received to imaging being performed (interval 2)
c) Total pre-analytical interval: time taken from imaging request being sent to test performed (intervals 1 + 2)
d) Reporting interval: time taken from test being performed to imaging report being issued (interval 3).
e) Total test interval: time taken from imaging request being sent to report being issued (interval 4 = 1 + 2 + 3)
f) Test-to-diagnosis interval: time from request being sent to cancer diagnosis made (interval 5 = 1 + 2 + 3+4)

Fig. A1.

Mean and Interquartile range of time interval for the testing phases for patients with an initial test 1–8 months pre-diagnosis.

Diagrammatic representation of the median number of days (in ovals) for each test interval
